# Toward Optimal Computation of Ultrasound Image Reconstruction Using CPU and GPU

**DOI:** 10.3390/s16121986

**Published:** 2016-11-24

**Authors:** Udomchai Techavipoo, Denchai Worasawate, Wittawat Boonleelakul, Rachaporn Keinprasit, Treepop Sunpetchniyom, Nobuhiko Sugino, Pairash Thajchayapong

**Affiliations:** 1 National Electronics and Computer Technology Center, Pathumthani 12120, Thailand; rachaporn.keinprasit@nectec.or.th (R.K.); treepop.sunpetchniyom@nectec.or.th (T.S.); pairash@nstda.or.th (P.T.); 2 Department of Electrical Engineering, Faculty of Engineering, Kasetsart University, Bangkok 10900, Thailand; fengdcw@ku.ac.th (D.W.); wittawat_1@yahoo.com (W.B.); 3 Department of Information Processing, Tokyo Institute of Technology, Tokyo 152-8552, Japan; sugino.n.aa@m.titech.ac.jp

**Keywords:** array transducer, CUDA, dynamic receive beamforming, graphics processing unit, image reconstruction, ultrasound imaging

## Abstract

An ultrasound image is reconstructed from echo signals received by array elements of a transducer. The time of flight of the echo depends on the distance between the focus to the array elements. The received echo signals have to be delayed to make their wave fronts and phase coherent before summing the signals. In digital beamforming, the delays are not always located at the sampled points. Generally, the values of the delayed signals are estimated by the values of the nearest samples. This method is fast and easy, however inaccurate. There are other methods available for increasing the accuracy of the delayed signals and, consequently, the quality of the beamformed signals; for example, the in-phase (I)/quadrature (Q) interpolation, which is more time consuming but provides more accurate values than the nearest samples. This paper compares the signals after dynamic receive beamforming, in which the echo signals are delayed using two methods, the nearest sample method and the I/Q interpolation method. The comparisons of the visual qualities of the reconstructed images and the qualities of the beamformed signals are reported. Moreover, the computational speeds of these methods are also optimized by reorganizing the data processing flow and by applying the graphics processing unit (GPU). The use of single and double precision floating-point formats of the intermediate data is also considered. The speeds with and without these optimizations are also compared.

## 1. Introduction

Ultrasound imaging using arrays of transducer elements has been widely applied in medicine [1,2] and industry [3,4]. One of its challenges is to deal with real-time applications. To improve the image qualities, many imaging techniques require more complex algorithms [5,6], possibly resulting in longer computational time and preventing them from being real-time. An ultrasound image is composed of many scanlines. Each scanline is created from beamforming of many echo signals, i.e., coherent delay-and-sum of echo signals received by transducer array elements. The transducer sends pulse signals and receives echo signals reflected from scatterers or interfaces inside an object before beamforming these echo signals to form a scanline on an image. These processes are repeated for different scanline locations to form the entire image. Generally, the beamforming adds delays to the signals received from the array elements. These delay values are calculated from the distances between the elements and a focal point on the scanline in order to equalize the wave fronts and the phases of the echo signals at that focus. This receive beamforming can be performed at many focal points on a scanline, e.g., dynamically beamforming at every point on a scanline. However, the delays need to be adjusted for each focus. Using inaccurate delays results in noisy beamformed signals [7].

At present, many ultrasound devices digitize the echo signals that come from the transducer array elements before beamforming. The required beamforming delays are hardly ever matched with the existing sampling points. Therefore, the subsample estimation of the delayed signal values among these sampling points is needed. The values of the nearest samples are usually used because of the fast processing. Using the values from the nearest samples is equivalent to using inaccurate delay times. This makes the delayed signals dephased, and the summation of these signals provides smaller magnitudes. Moreover, the delayed signals could be dephased further if the sampling rate is insufficient. For example, if the sampling rate is less than two times of the carrier frequency, two consecutive samples are separated more than a half of carrier period apart, and therefore, the nearest samples could take the values that their phase difference is one π radian. This makes the summation of the magnitudes go to zero instead of the maximum. Another subsample estimation uses the in-phase (I) and quadrature (Q) components of the echo signals. If the system has a sampling rate equal to four (or a multiple of four) times the pulse center frequency, these I/Q components can be found easily using a quarter wavelength shift. These I/Q components can be used to calculate the values of the delayed signals using phase rotational circuitry [8]. However, if the sampling rate is not equal to four (or a multiple of four) times the pulse center frequency, the generalized sub-sample delay method [9] can be applied. It calculates the Q components using trigonometric functions of the angular deviation between the existing samples and the exact quarter wavelength shift. This I/Q interpolation method provides better estimates than the nearest sample method, but extra processing time is required for its complex algorithm.

Researchers have applied field programmable gate arrays (FPGA) for beamforming in order to increase the processing speeds. A single FPGA has been used in an ultrasound imaging system in order to meet the high processing requirements of the beamforming [10,11]. The beamforming algorithms need to be suitably optimized for the FPGA in terms of the memory size, the number of the logic gates and the data transferring speed to/from the FPGA. For this reason, the delays are generally precomputed and kept in the FPGA memory to save computational time and logic gates. The number of focal points is also reduced to save the FPGA memory, e.g., instead of focusing on every point on the scanline, the scanline is divided into windows, and each window has only one focal point. Moreover, the size of the windows also increases along the depth to further reduce the focal points [12]. Because of using one focal point per window, other points in that window are out of focus. The out-of-focus delay error can be compensated using precomputed linear line approximation [13,14]. These methods are the so-called pseudo-dynamic receive beamforming. The proper adjustment of the algorithms for FPGA is quite a challenge because of the dependence of the hardware design and the FPGA resources. Moreover, the development on FPGA requires experienced programming skill on specific languages, such as Verilog or VHDL, which take time for the development.

Graphics processing units (GPU) have been used for graphic processing and other applications that need computational speed. This has developed from a fixed-function special-purpose processor into a full-fledged parallel programmable processor with fixed-function special-purpose functionality [15]. Its programming environment has been changed from graphics application program interfaces (API) to high-level language interfaces, such as NVIDIA’s compute unified device architecture (CUDA), with which general developers are already familiar. Currently, it has been used in ultrasound beamforming [16,17,18,19]. The plane wave compounding and synthetic aperture (SA) imaging computations have been speeded up by an array of two GPUs; one is used to analyze and beamform the echo signals to generate low resolution images, and the other is used to compound those results to a high resolution image [16]. Another SA-based algorithm using low numbers of transmit and receive elements (one and two, respectively) is also accelerated using a GPU [17,18]. There are four processes, i.e., band-pass filtering, beamforming, envelope detection and display, where the GPU executes each process at a time by using all available GPU cores in parallel. Moreover, a GPU is also used to estimate the depth-dependent frequency of the pulse before applying the results to demodulate the beamformed radiofrequency (RF) signals [19].

In this paper, the data processing flow for ultrasound image reconstruction is reorganized to reduce the computational time. The improvement of the beamformed signal quality after using the I/Q interpolation method for subsample estimation instead of using the nearest samples has been investigated to demonstrate the tread-off between the signal quality and the computational time caused by the algorithm complexity. This investigation includes programming using the CPU and GPU for the calculation to find suitable implementation recommendations. The beamforming using the I/Q interpolation method in which the sampling frequency is not equal to four times the pulse center frequency is compared to the nearest sample method. The output signals after beamforming using these two methods are compared to our reference beamforming results obtained using upsampled echo signals with a high sampling rate. The mean squared errors of the beamformed signals between the reference and our methods are compared. The visual qualities of the ultrasound images and the quality of the beamformed signals reconstructed using these methods are compared.

This paper is extended from the preliminary work in [20]. Further optimization of the computer programs has been done. The direct convolution instead of the Fourier transform method for DC cancellation has been investigated and used to improve the computational time. In addition, more experimentations in different computer platforms, 16-bit and 32-bit, of the compiled programs and in different precisions of the floating-point (FP) formats, single precision (FP32) and double precisions (FP64) for the intermediate data have been explored to optimize the computational time. Furthermore, the number of repetitions and the number of experimental datasets have increased to enhance the conclusion.

## 2. Materials and Methods

### 2.1. Ultrasound Imaging System

**Figure 1:**
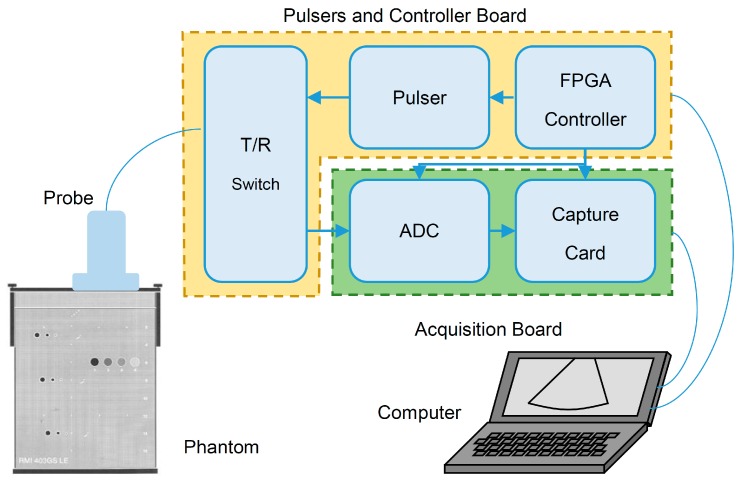
The schematic of our ultrasound imaging system with a tissue-mimicking phantom and a computer. T/R, transmit/receive.

Our ultrasound imaging system [21] is composed of three parts. The first part is the ultrasound probe used for sending pulses and receiving echo signals. It is a 7.5-MHz linear probe with 80 elements and a 0.5-mm element spacing. The second part is the pulsers and controller board. It is composed of 32 transmit/receive (T/R) switches, 32 pulsers and an FPGA controller. The controller receives commands from a computer via an RS232 port. The third part is the acquisition board. It contains an analog-to-digital convertor (ADC) card (8 channels, 12-bit and 40-MHz sampling rate) and a capture card. It digitizes the echo signals and transfers the data to a computer via USB 2.0. The schematic of the system is shown in Figure 1. The ADC contains on-chip selectable −3-dB cutoff frequencies of high-pass and low-pass filters. For our experiment, these cutoff frequencies are set at 3.73 to 11.56 MHz, suitable for the 7.5-MHz probe center frequency.

### 2.2. Experimental Data

The digitized echo signals from each array element of the probe are acquired from our ultrasound imaging system [21]. Thirty-two consecutive array elements are used to transmit pulses for a scanline. The transmit focusing is set at a 6-cm depth. The same set of array elements is then used to receive the echo signals. The echo signals are digitized and transferred to the computer. Then, they are offline beamformed on the computer. A precision multi-purpose phantom (403GS LE, Gammex Inc., Middleton, WI, USA) is used in our experiment. Three experiments are performed by scanning this phantom at 3 locations: (1) its anechoic target and ±6-dB grey scale targets; (2) its ±6-dB grey scale targets and high scatterer target; and (3) its pin targets. The sound speed in the material is 1540 m/s. Eighty-one scanlines are collected, and each scanline contains 8192 samples. The total data size is 42.5 Mbytes (=8192 samples × 32 receive signals × 81 scanlines × 2 bytes per sample).

### 2.3. Image Reconstruction Algorithms

**Figure 2:**
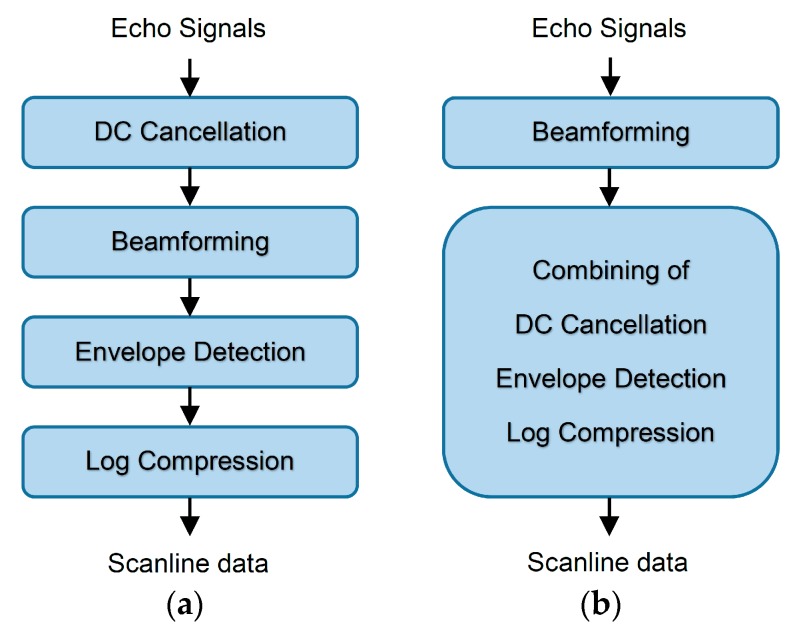
Block diagrams of the processes used for the ultrasound image reconstruction in the conventional (**a**) and the proposed (**b**) methods.

Block diagrams of a conventional and our image reconstruction algorithms are shown in Figure 2. The conventional algorithm in Figure 2a is composed of four processes, DC cancellation, beamforming, envelope detection and log compression. Our proposed algorithm in Figure 2b is modified from the conventional one by switching the DC cancellation to be after the beamforming and by combining it with the envelope detection and log compression. The switching could be done by assuming that the DC cancellation and the beamforming are linear operations. This switching reduces the number of signals to be filtered from 32 to 1, thus reducing the computational time. The process details for each block in Figure 2 are as follows.

#### 2.3.1. DC Cancellation

A high-pass filter is applied for removing the DC components from the echo signals. It is an equiripple linear-phase FIR filter of length 11. It is designed using MATLAB (MathWorks Inc., Natick, MA, USA) to have a 0.1-MHz stopband with −50 dB of attenuation and a 7-MHz passband with a 0.1-dB ripple. Even though the ADC chip has a band-pass filter as described earlier, there still exists small DC offset around −2 to 18 digital output levels (around a −2.7 to 23.9-µV input at a 51.3-dB gain) remaining in the signals. This DC component is, therefore, removed in this stage.

This high-pass filter can be applied to the echo signals by (1) using direct convolution in the time domain or (2) using the fast Fourier transform (FFT) to multiply the frequency components in the frequency domain and then using the inverse Fourier transform to transform them back to the time domain. Precomputed frequency components of the filter are used. These frequency components are calculated by zero padding the filter coefficients to have the same length as of the signals before applying the Fourier transform. These two approaches are tested to find the optimum computational time. The results are shown in the next section.

#### 2.3.2. Beamforming

The delay-and-sum beamformer is used. Two interpolation methods are compared, the nearest sample method and the I/Q interpolation method with the sampling frequency not equal to 4 times (or a multiple of 4 times) the pulse center frequency [9].

The nearest sample method simply selects the sample, *s_nearest_*, that is closest to the needed delay time, i.e.,

(1) $$s_{nearest}=S\left(t\right){|}_{t=n_{nearest}T_{s}},$$

(2) $$n_{nearest}=\mathrm{round}\left((t_{ref}+\tau \right)/T_{s})),$$

where *S*(*t*) is the echo signal; *n_nearest_* is the number of samples closest to the needed delay; *t_ref_* is the reference time at the acquired depth of the scanline, i.e., *t_ref_* = *d/c*, where *d* is the depth and *c* is the sound speed in tissue equal to 1540 m/s; *τ* is the needed real delay time; and *T_s_* is the sample period equal to 1/*f_s_*, where *f_s_* is the sampling frequency.

The I/Q interpolation method selects two samples, one is the nearest sample, *s_nearest_*, as described above, and the other is the closest sample to the quadrature shift from the nearest sample, *s_Q-shift_*. This quadrature shift is equal to 0.25*T_c_*, where *T_c_* is the period of the pulse center frequency and equal to 1/*f_c_*, where *f_c_* is the carrier frequency. This *s_Q-shift_* can be calculated as:

(3) $$s_{\mathrm{Q-shift}}=S\left(t\right){|}_{t=\left(n_{nearest}+n_{\mathrm{Q-shift}}\right)T_{s}}$$

(4) $$n_{\mathrm{Q-shift}}=\mathrm{round}\left(0.25\;T_{c}/T_{s}\right)$$

where *n_Q-shift_* is the number of samples closest to the quadrature shift. The I/Q interpolated value, *s_I/Q_*, can be calculated by:

(5) $$s_{I/Q}=s_{nearest}\left[\cos\left(a\right)+\sin\left(a\right)\tan\left(e\right)\right]+s_{\mathrm{Q-shift}}\left[\sin\left(a\right)\sec\left(e\right)\right]$$

(6) $$a=2\pi f_{c}\left(t_{ref}+\tau -n_{nearest}T_{s}\right),$$

(7) $$e=2\pi f_{c}\left(n_{\mathrm{Q-shift}}T_{s}-0.25T_{c}\right)$$

where *a* is the angular remaining to the real delay value; and *e* is the angular shift error between the quadrature shift and its nearest. For our ultrasound imaging system, where *f_c_* = 7.5 MHz and *f_s_* = 40 MHz, *n_Q-shift_* obtained from Equation (4) is equal to 1 sample, and *e* obtained from Equation (7) is approximately equal to −0.3927 radians.

The delay times for each depth can be precalculated for all echo signals and for all scanlines, i.e., 32 echo signals and 81 scanlines. Note that the nearest sample method uses the delay times in the integer number of samples, while the I/Q interpolation method uses the delay times in FP numbers of samples. For our experiments, a 4-byte integer (*int*) is used for the nearest sample method, and both FP64 (*double*) and FP32 (*float*) are used in the I/Q interpolation method. The total memory sizes to keep these delay times are around 85 MBytes for the integers and the FP32s and 170 MBytes for the FP64s.

#### 2.3.3. Envelope Detection

The Hilbert transform is used for the envelope detection. It is done by applying the Fourier transform, modifying the frequency components and applying the inverse Fourier transform [22]. The signal envelope, *Env*, can be calculated from, $Env=sqrt\left(hr^{2}+hi^{2}\right)$, where *hr* and *hi* are the real and the imaginary parts of the Hilbert transform, respectively, and sqrt() is the square root operation. However, in order to reduce the computational time, the square root operation is not performed, and therefore, the envelope square, *Env*^2^, is kept and used for the next step.

#### 2.3.4. Log Compression

This process is used to adjust the dynamic range of the envelope before displaying on the monitor. The 10-based logarithmic function is used to adjust the envelope into decibel units (dB), i.e., $Env_{dB}=10\log\left(Env^{2}\right)$. The envelope square is from the previous step. Note that to protect the undefined condition of log(*x*) when *x* ≤ 0, the inputs are truncated to be greater than 10^−12^ before being passed to this function.

#### 2.3.5. Combining of DC Cancellation, Envelope Detection and Log Compression

Since the DC cancellation (using high-pass filtering) and the envelope detection (using the Hilbert transform) are performed in the frequency domain, therefore combining them together reduces the computations of the Fourier and the inverse Fourier transforms. This is done by precalculating the frequency components of the high-pass filter and premultiplying them to the Hilbert transform frequency components. These results are kept in the memory and ready to be applied to the frequency components of the beamformed signals. After the inverse Fourier transform, the magnitudes of the signals are calculated and immediately log compressed to save computational time.

### 2.4. Beamformed Signal Comparison

To evaluate the performance of the proposed ultrasound image reconstruction method, the RF signals after being beamformed are compared to the reference beamformed signals in terms of the mean square errors (MSE). The reference beamformed signals are created by using the FP64 and by the following: (1) upsampling the digitized echo signals 20 times the 40-MHz sampling frequency, i.e., 800 MHz; (2) DC cancelling or high-pass filtering the upsampled echo signals; and (3) beamforming the results using the nearest sample method. The sampling frequency of 800 MHz is assumed to be high enough such that the nearest sample is almost equal to the needed delayed signals. For each scanline, the RF signals corresponding to the depth between 4 cm and 8 cm are used for the comparisons. This depth covers the greyscale targets of the phantom and provides a general representation of the ultrasound signals.

There are 8 combinations of the following schemes to be tested: the conventional and the proposed methods, the nearest sample and the I/Q interpolation methods and the FP64 and FP32 formats. The final images from these results are also compared in terms of visual qualities. All of these computations are performed on MATLAB (MathWorks Inc., Natick, MA, USA) without recording the computational time.

### 2.5. CPU and GPU Programming

The computer used in this paper is a laptop computer (G750JW, Asustek Computer (Thailand) Co., Ltd., Bangkok, Thailand) with the Windows 8 64-bit operating system. The computer specification is as follows: CPU (CORE i7-4700HQ, Intel Corporation Co., Ltd., Santa Clara, CA, USA), 2.40-GHz clock speed, 4 cores and 8-GB DDR3 RAM. This laptop contains a graphics card (GeForce GTX 765M, NVIDIA Corporation Co., Ltd., Santa Clara, CA, USA). The GPU specification is as follows: 3.0 compute capability, 2048 MBytes global memory, 768 CUDA cores, 863-MHz GPU clock rate, 2004-MHz memory clock rate, 128-bit memory bus width and 64-GB/s memory bandwidth. The maximum number of threads per block is 1024. The programming is done using visual C++ with CUDA library Version 6.5.14. The Fourier and inverse Fourier transforms on the CPU are computed using the *Fastest Fourier Transform in the West* (FFTW) library Version 3.3.5 [23], while those on the GPU are done using the NVIDIA CUDA Fast Fourier Transform library, i.e., cuFFT.

The algorithms for the CPU and the GPU are very similar. The processes in the blocks in Figure 2 are sequentially executed as shown. The CPU or the GPU dedicates all resources to compute each block at a time. For the GPU, all digitized echo signals are uploaded from the host computer to the device GPU’s global memory at the beginning, and the results after the envelope detection are downloaded from the GPU back to the computer.

Beamforming is an embarrassingly parallel problem, which is easy to separate into parallel tasks. The digitized echo data or the raw data can be considered as 3-D data that are composed of 8192 samples × 32 signals × 81 scanlines. They need to be reconstructed into 2-D data or beamformed data of 8192 samples × 81 scanlines.

**Figure 3:**
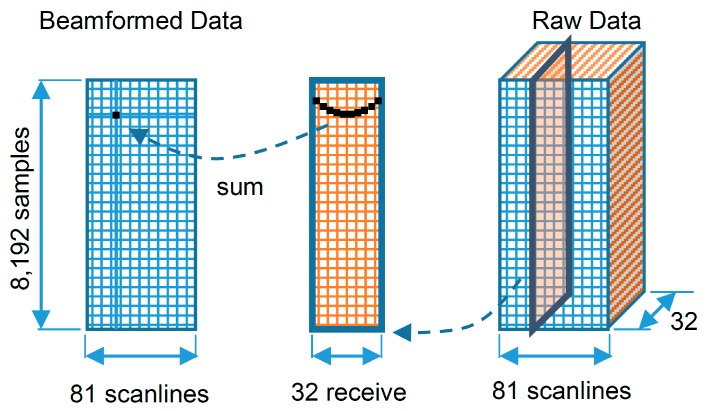
The data flowchart for beamforming.

The data flowchart for computing beamforming is shown in Figure 3. The program starts iteratively executing the first to the last (8192nd) samples of the first scanline. Then, the program shifts to execute the next scanline until finishing the last (81st) scanline. For the CPU, the for-loops are used to find the result values of the samples, executing one sample at a time until the last sample. In contrast, the GPU eliminates the outer for-loop and uses its 768 CUDA cores to execute 768 samples in parallel. In Figure 3, to evaluate the value of a sample in the beamformed data (on the left), dynamic focusing is performed by selecting samples from the 32 receive signals (on the center) depending on the required delay times. These 32 receive signals are parts of the raw data (on the right). These delay times are precalculated and uploaded to the GPU’s global memory at the beginning. The nearest sample and the I/Q interpolation methods, already explained in the previous section, are then performed with summation or interpolation on these selected points.

The processing time of each block is recorded using a simple function clock() for the CPU program and using the following functions, cudaEventCreate(), cudaEventRecord(), cudaEventSynchronize() and cudaEventElapsedTime(), for the GPU program. These processing times versus the computer platforms of 32-bit and 64-bit are investigated, as well as those versus the FP64 and FP32 formats.

The programs measure the computational times for using all combinations of the following: the conventional and the proposed methods, the nearest sample and the I/Q interpolation methods, the CPU and the GPU, the 32-bit and the 64-bit platforms of the compiled programs and, finally, the FP64 and the FP32 formats. The programs are compiled and run in release mode for 500 repetitions, and their computational times for the processes (in the flowcharts in Figure 2) are recorded. The uploading and downloading of data between the host computer and the device GPU are also recorded.

## 3. Results and Discussion

**Figure 4:**
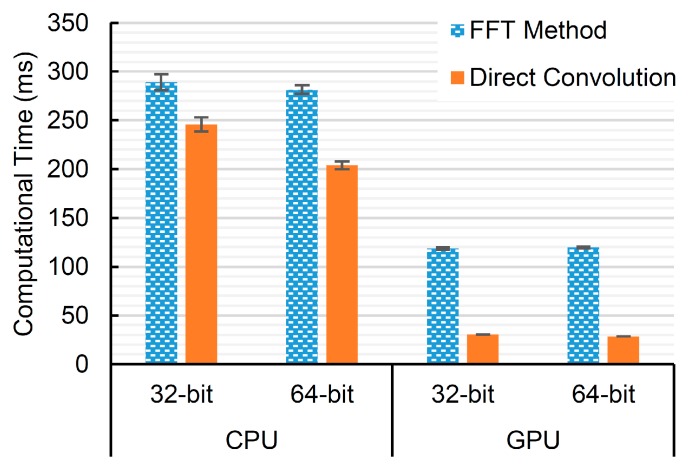
The computational times of applying an 11-point high-pass filter on 2592 signals (from 32 Rx elements × 81 scanlines) of 8192 samples using the Fourier transform method and direct convolution. These methods are executed on different computer platforms of 32 and 64 bits and using the CPU and the GPU. The error bars are one standard deviation over 100 repetitions

The computational times for computing high-pass filtering for the DC cancellation using the Fourier transform method and the direct convolution are shown in Figure 4. This computations are performed using the FP64. Surprisingly, the direct convolution is faster than the Fourier transform method for all cases. These gains are around 1.2- to 1.4-times on the CPU and around 3.9- to 4.7-times on the GPU. Even though the FFT method has O(n log n) complexity that is better than O(n^2^) of the direct convolution, it has been shown in the literature [24,25] that for short lengths of filters and signals, i.e., a number of samples less 128 points, direct convolution is significantly faster than the FFT method because of the lower numbers of real multiplications. Moreover, it has been reported that, on GPUs, the length of the filters can be much longer because of the intensively parallel computation. Note that the program has to be compiled and run in release mode to see this advantageous outperform of the direct convolution. Running in debug mode, the performance of the direct convolution is very poor compared to the FFT method that already uses precompiled libraries of the FFTW or cuFFT. For this reason, the direct convolution is consequently used in the experiments to compare the processing times.

**Figure 5:**
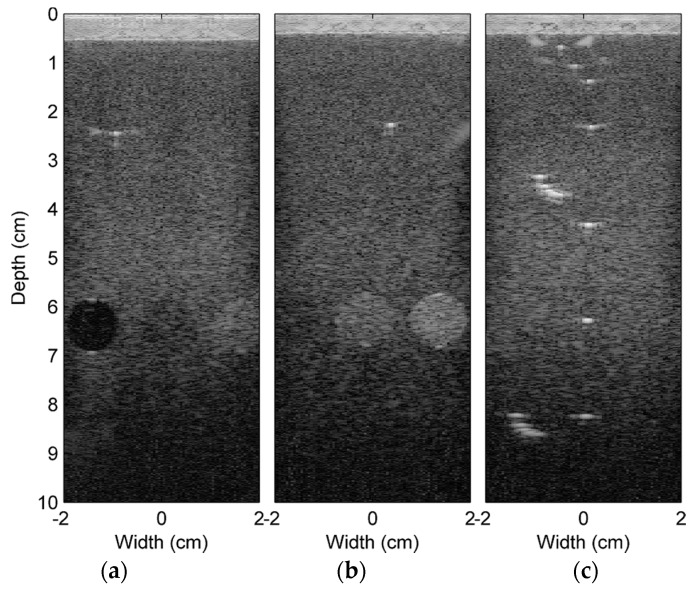
Ultrasound image references scanned at different locations of the precision multi-purpose phantom that are used in Experiments 1–3 (**a**–**c**), respectively.

Figure 5 demonstrates the ultrasound images reconstructed using the reference beamformed signals. These images are displayed in a dB scale ranging from 0 to −80 dB. This range is also used for all ultrasound images displayed here. As described previously, these reference signals are created by upsampling the experimental signals into an 800-MHz sampling rate and using the nearest sample method for estimating the delayed signals in beamforming. These signals are from Experiments 1 to 3, shown in Figure 5a–c, respectively, and are taken from different locations of the phantom. As shown, Figure 5a contains three grey scale cylindrical targets that are, from left to right, an anechoic target representing a fluid-filled cyst and two grey scale targets of −6 dB and +6 dB compared to the background. Figure 5b also contains three grey scale targets that are, from left to right, −6 dB, +6 dB and high scatterer targets. Figure 5c contains pin targets that provide a very high amplitude of the echo signals. The pins are at different depths and in different combinations. The purposes of these pin targets are for dead-zone measurement, resolution measurement and vertical distance calibration. These grey scale targets and pin targets should provide comparable objects for general ultrasound signals.

**Figure 6:**
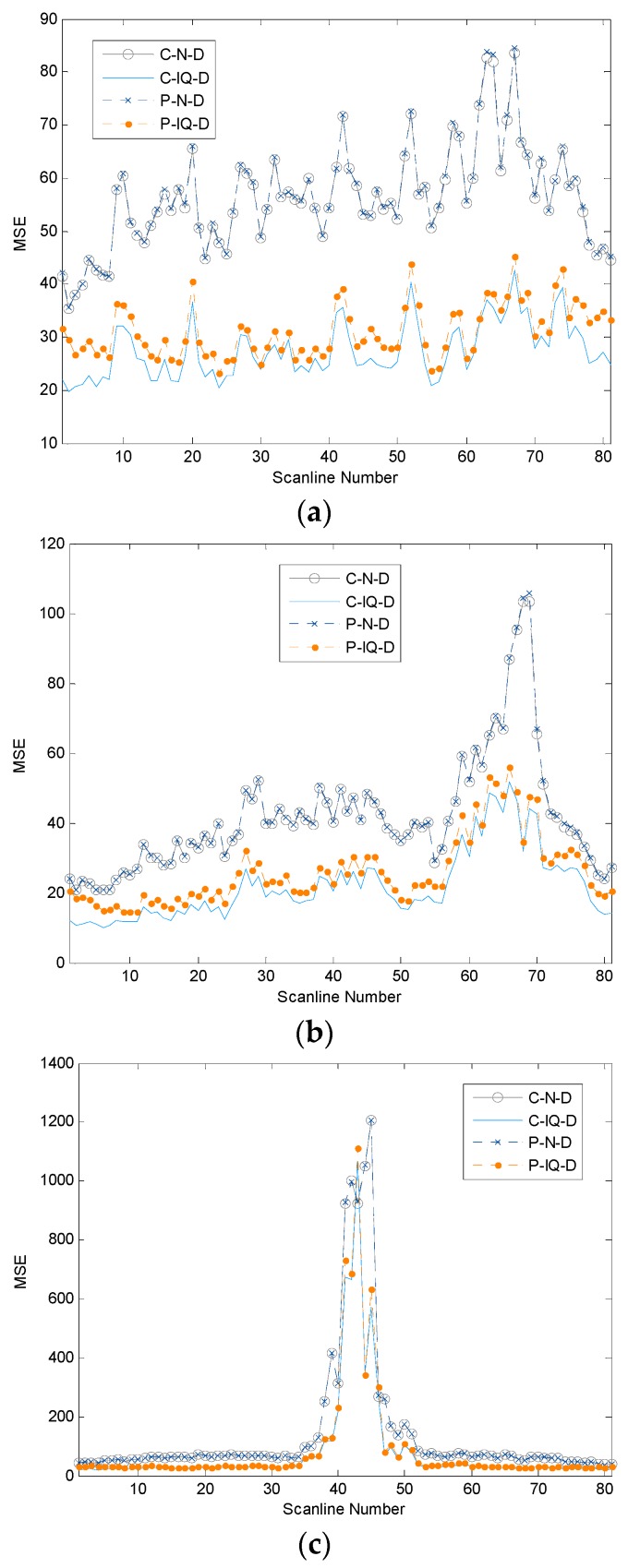
MSEs of all scanlines between the reference beamformed signals and the signals beamformed using four different schemes, conventional (C)-nearest sample (N)-double precision floating format (D), C- in-phase/quadrature (IQ)-D, proposed (P) method-N-D and P-IQ-D. The figures are from Experiments 1 to 3 (**a**–**c**), respectively.

Figure 6 plots the MSEs between the reference beamformed signals and the beamformed signals using different methods for all scanlines (Scanline Numbers 1 to 81) and for all experiments (Experiment 1 to 3). The results from Experiments 1 to 3 are in Figure 6a–c, respectively. The results are all from double precision floating format (FP64 or D). However, they are from different combinations of the conventional (C) and the proposed (P) methods and the nearest sample (N) and I/Q interpolation (IQ) methods, e.g., C-N-D, C-IQ-D, P-N-D and P-IQ-D. In general, the MSEs from C-N-D and P-N-D are very similar, i.e., the plots are almost on top of each other. These MSEs are higher (poorer) than those from C-IQ-D and P-IQ-D. These imply that the beamformed signals from the I/Q interpolation method are closer to the reference signals than those of the nearest sample method. Note that the MSEs from the single precision floating point format (FP32 or S) are very similar to these results and are not shown here. The difference of the MSEs for each scanline between the results using the FP64 and the FP32 is within ±1.2 units of the MSE.

For using I/Q interpolation methods, the MSEs from the P-IQ-D method are slightly higher (poorer) than those from the C-IQ-D. The difference comes from the change of the order of the high-pass filtering to remove the DC components from the position before beamforming to the position after beamforming. Even though this change reduces the number of signals to be filtered, i.e., from 32 signals to one signal, the quality of the beamformed signals is slightly poorer.

We also notice that the high MSEs (poor beamformed signal quality) occur at the scanlines that have higher echo signals, e.g., the Scanline Numbers 60 to 70 from Experiment 2 in Figure 6b corresponding to the high scatterer grey scale target in Figure 5b and the Scanline Numbers 35 to 50 from Experiment 3 in Figure 6c corresponding to the proximity of the pin targets in Figure 5c. This should be caused by the fact that slight delaying of these high amplitude signals creates a large change of the signal values.

**Figure 7:**
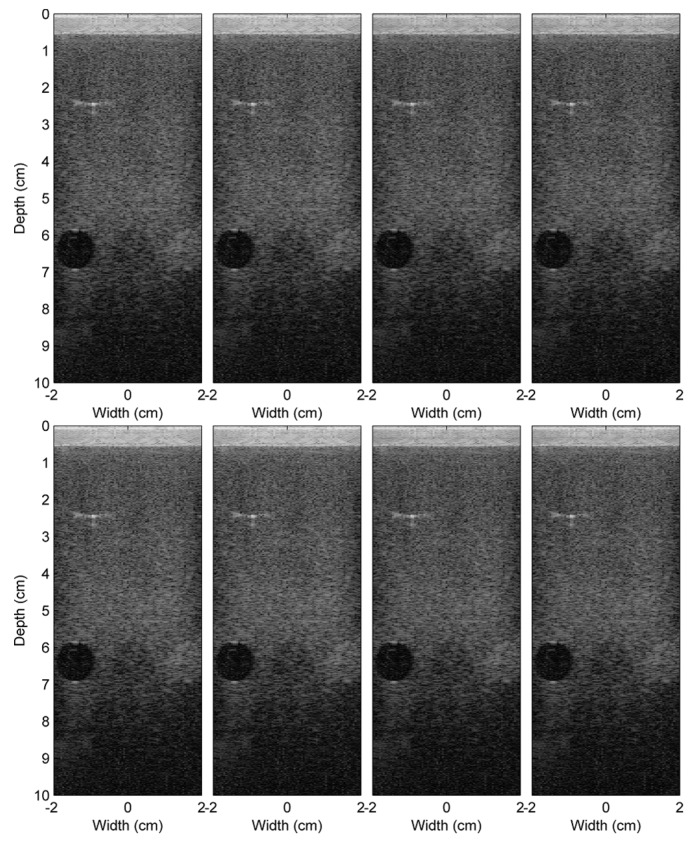
Comparison of the ultrasound images (from Experiment 1) reconstructed using eight different schemes, C-N-D, C-IQ-D, P-N-D, P-IQ-D, C-N-single (S) , C-IQ-S, P-N-S and P-IQ-S, from the left to the right and from the top to bottom, respectively.

**Figure 8:**
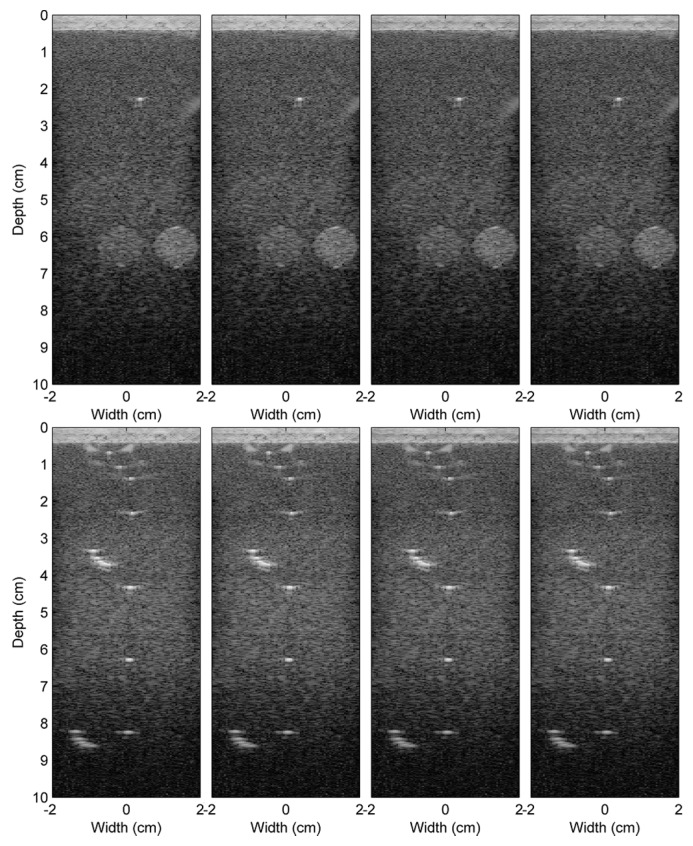
Comparison of the ultrasound images from Experiments 2 (top row) and 3 (bottom row) reconstructed usingfour4 different schemes, C-N-D, C-IQ-D, P-N-D and P-IQ-D, from the left to the right, respectively.

The reconstructed images from the beamformed signals from different schemes of Experiment 1 are shown in Figure 7. As mentioned in the previous section, the different schemes are the different combinations of the following: the conventional (C) and the proposed (P) methods; the nearest sample (N) and the I/Q interpolation (IQ) methods; and the double (D) and the single (S) precision FP formats. Note that the image reconstructed using the reference beamformed signals of Experiment 1 is shown in Figure 5a. In general, all images are very similar. Although the RF beamformed signals from the I/Q interpolation method are closer to the reference, after passing these beamformed signals to the envelope detection and log compression, this advantage could not be visually noticeable on the reconstructed images. However, for other applications, such as elastography [26], which needs the phase information of the RF signals, this advantage might be revealed. The reconstructed images from Experiments 2 and 3 are shown in Figure 8. Only the results from the double precision floating point format are shown here. These images and the reference images in Figure 5b,c are very similar.

**Figure 9:**
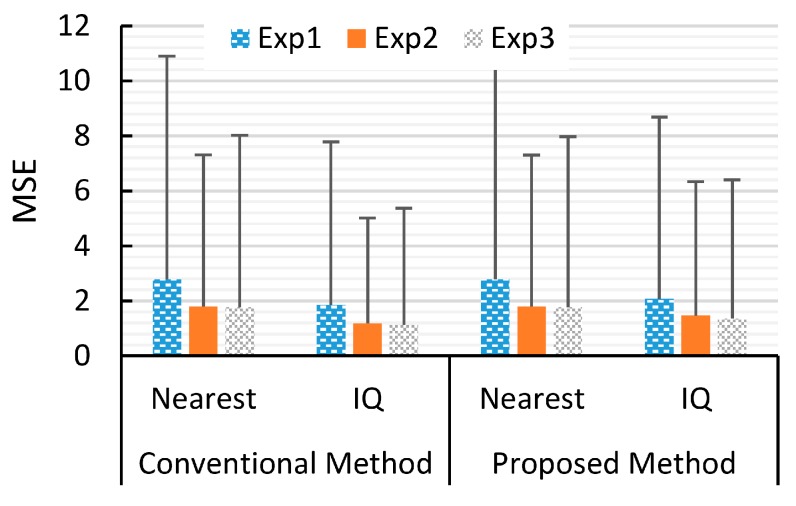
The bar graph of the MSEs between the reference ultrasound images and the reconstructed images using four different combinations of schemes: the conventional and proposed methods; and the nearest sample and the I/Q interpolation method. The upper error bars show one standard deviation of the squared errors. Note that the range of the ultrasound images is from 0 to −80 dB.

The MSEs and their standard deviations between the reference ultrasound images in Figure 5 and the images in Figure 7 and Figure 8 are shown in Figure 9. Only the results using FP64 are shown. As can be seen, the MSEs from all experiments are less than 2.8, and the standard deviations are less than 8.1. The pixel value of these images are in dB ranging from 0 to −80 dB. This confirms that the errors between the reference ultrasound images and the images from different reconstruction schemes are very similar.

The time for uploading the digitized echo data to the GPU is around 8.31 ms (for 42.5 MBytes = 8192 samples × 32 receive signals × 81 scanlines × 2 bytes of *short int*). The time for downloading the results from the GPU is around 1.14 and 0.61 ms, respectively, for the FP64 format (5.3 MBytes = 8192 samples × 81 scanlines × 8 bytes of *double*) and for the FP32 format (2.65 MBytes = 8192 samples × 81 scanlines × 4 bytes of *float*). These times are averaged from the recorded times of all tested schemes.

**Figure 10:**
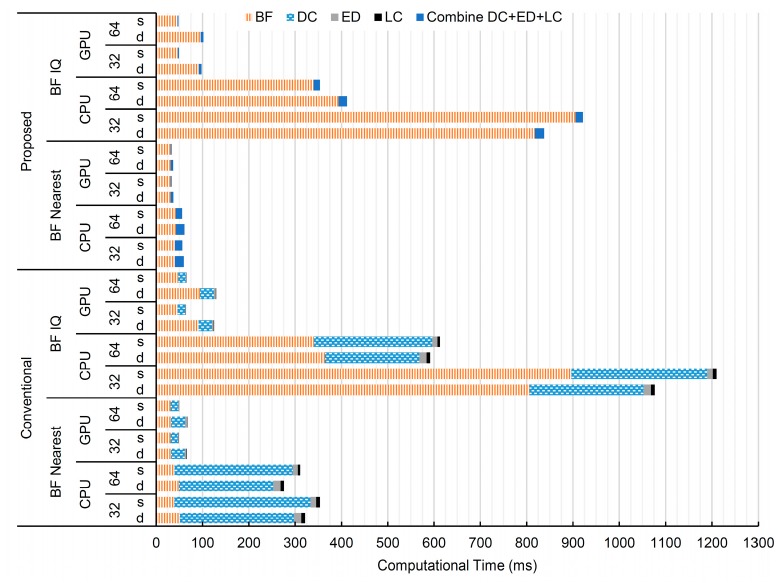
The mean of the computational time for ultrasound image reconstruction using different combinations of the following: the conventional and the purposed methods, the beamforming using the nearest sample and that using I/Q interpolation methods, the 32-bit and the 64-bit computer platforms and the double (D) and the single (S) precision FP formats. Notes: BF = beamforming, DC = DC cancellation, ED = envelope detection and LC = log compression.

The processing times for each block in Figure 2 using different schemes of the ultrasound image reconstruction are plotted in Figure 10. The details are also shown in Table 1. This figure demonstrates many aspects as follows:

1. The purposed method, which switches the DC cancellation and the beamforming, as well as combines the DC cancellation with the envelope detection and log compression, greatly improves the computational time compared to the conventional method. As can be seen, the DC cancellation processing time has almost disappeared from the plot.
2. Beamforming using the I/Q interpolation method is slower than that using the nearest sample method. Using the CPU, the gaps are quite large, around 15-times slower. However, using the GPU, the gaps are no more than two-times slower. For the nearest sample method, the best performance in computational time ranges from 31.09 to 31.13 ms using the proposed method and the GPU and any of the 32-bit or 64-bit program platforms or the FP32 or the FP64 formats. For the I/Q interpolation, the best performance is at 45.75 ms using the purposed method, the GPU, the 64-bit platform and the FP32 format.
3. Using the GPU greatly reduces the processing time. As expected, the GPU parallel computes the tasks using threads, and our tasks can be pleasingly separated into independent threads. Using the GPU needs extra time to upload and download the data between the host and the device, which is quite fast around 8.31 ms for uploading and 1.14 or 0.61 ms for downloading FP64 or FP32 data, respectively. Moreover, these memory transfers between host and device can be performed concurrently with other GPU processes [27].
4. Using a 64-bit computer platform program instead of 32-bit improves the computational time impressively and significantly for the schemes using the CPU with the I/Q interpolation method. For other schemes, the results are slightly better. This could be the fact that our testing CPU is an Intel CORE i7, which is a 64-bit CPU [28], which matches with the 64-bit compiled program. Moreover, the I/Q interpolation method requires many arithmetic calculations, compared to the nearest sample counterpart. This makes the advantage be more pronounced in the I/Q interpolation method than the nearest sample method. For the GPU, changing from 32-bit to 64-bit gives slight improvement since most of the calculations are performed on the GPU that has its own instructions independent of the computer platform compilation.
5. Using the FP32 format instead of the FP64 provides advantages on the GPU, but slightly and only for some processes and schemes on the CPU. It is certain that using the FP32 format saves memory space and the time to copy the memory between the host computer and the device GPU. Other factors are based on the hardware that intrinsically supports the FP64 computation. For the GPU, the tested Geforce GTX765M supports both FP64 and FP32 computation. However, the number of FP32 units is higher than that of FP64 units. Based on the Kepler (GK106) microarchitecture, the unit ratio of the FP64/FP32 is 1/24 [29]. For this reason, using the FP32 format reduces the computational time. For the CPU, the tested programs are not customized for the Intel CORE i7, which provides instruction sets for the single instruction, multiple data (SIMD) parallel computing, i.e., using multiple processing elements to perform the same operation on multiple data points simultaneously and also known as the streaming SIMD extensions (SSE). These instruction sets provide abilities to perform an instruction on two FP64s or four FP32s simultaneously. The tested program applies only the standard FP units (FPU) that support both FP32 and FP64 data. The data loaded from the memory into the FPU are automatically converted into a double extended-precision FP format on 80-bit registers [30]. The results after operations are converted back into a shorter FP format and transferred back into the memory. For this reason, using the FP32 does not improve the computational time.
6. The best performance for total image reconstruction time is at 33.23 ms for using the GPU, the proposed method, the nearest sample method, the 64-bit platform and the FP32 format, excluding the uploading/downloading data. If the CPU is only the option, the best performance is at around 56.06 ms using the proposed method, the nearest sample method, the 64-bit platform and the FP32 format. These could provide the frame rate at 30.1 and 17.8 frames/s for the GPU and the CPU, respectively. If the I/Q interpolation is needed, the best performance is at 47.89 ms using the proposed method, the GPU, the 64-bit platform and the FP32 format. This gives the frame rate around 20.9 frames/s. Note that for our GPU programs, the shared memory is not used yet. All raw data, delay tables and other intermediate variables are all defined in the global memory. It is faster to access the shared memory than the global memory. A better algorithm that utilizes the shared memory is still needed. However, the size of the shared memory is limited (48 kBytes), and the memory management to avoid bank conflict, i.e., accessing many data from the same memory bank and making the access to be done in serial instead of parallel, is challenging.

## 4. Conclusions

It has been shown that the I/Q interpolated beamforming provides a higher quality of the RF scanlines than the nearest sample beamforming for all scanlines of all tested images. However, its calculation is more complex and takes more time to be computed, e.g., 6.3–16.3 times and 1.4–2.8 times using the CPU and the GPU, respectively, in our experiments. Moreover, this higher quality of the RF scanlines does not reflect the visual qualities of the B-mode images. It might benefit other applications that need RF scanline phase information, such as elastography. In addition, we have also demonstrated that the GPU certainly improves the computational speed for ultrasound beamforming and image reconstruction. Its overheads of the data transfers between the host computer and the device GPU are short and can be pipelined, thus unaffecting the imaging frame rate throughput. For our graphics card having 768 CUDA cores, the frame rate using the nearest sample method could be 30.1 frames/s, which is more than the requirement of 25 frames/s for real-time imaging. However, the frame rate using I/Q interpolation could be only 20.9 frames/s and not enough to be a real-time application. To solve this problem, a higher performance GPU is needed, e.g., a GPU with higher numbers of CUDA cores, such as 1536 or 3072 cores, which are already available in modern graphics cards. Rather than increase the CUDA cores, reducing the beamform dynamic focusing points [12] or applying pseudo-dynamic beamforming [13] could be an economic solution.

## Figures and Tables

**Table 1:** The mean and the standard deviation of the computational time (ms) for ultrasound image reconstruction using different schemes calculated over 500 repetitions.

Ultrasound Processes		BF Nearest								BF IQ							
		CPU				GPU				CPU				GPU			
		32		64		32		64		32		64		32		64	
		d	s	d	s	d	s	d	s	d	s	d	s	d	s	d	s
		**Conventional Method**															
**BF**	**mean**	50.69	38.86	48.92	39.38	32.12	31.08	32.14	31.05	805.95	896.12	364.28	340.86	91.17	46.41	94.80	47.56
	**sd**	6.71	7.87	5.28	7.83	0.034	0.030	0.031	0.029	8.39	8.74	9.31	6.77	0.036	0.039	0.034	0.037
**DC**	**mean**	246.82	294.80	203.93	255.41	29.35	16.28	30.26	16.58	246.91	293.72	203.96	255.36	29.14	16.20	29.90	16.46
	**sd**	6.82	5.54	3.75	7.44	0.036	0.034	0.023	0.028	6.45	6.50	3.68	7.50	0.028	0.022	0.041	0.024
**ED**	**mean**	15.66	11.82	15.74	11.24	3.85	1.20	3.90	1.21	15.66	11.88	15.71	10.95	3.84	1.19	3.90	1.21
	**sd**	1.29	6.73	1.30	7.03	0.036	0.049	0.025	0.017	0.47	6.69	1.31	7.18	0.031	0.020	0.027	0.016
**LC**	**mean**	8.12	8.46	7.45	4.98	0.98	0.14	0.99	0.14	8.24	8.30	7.78	5.88	0.98	0.14	0.99	0.14
	**sd**	7.82	7.79	7.80	7.28	0.001	0.005	0.001	0.003	7.78	7.77	7.82	7.59	0.001	0.003	0.001	0.004
**Total**		**321.29**	**353.94**	**276.03**	**311.01**	**66.29**	**48.69**	**67.30**	**48.99**	**1076.76**	**1210.01**	**591.72**	**613.04**	**125.13**	**63.94**	**129.59**	**65.37**
		**Proposed Method**															
**BF**	**mean**	40.13	40.19	42.37	42.17	31.13	31.13	31.09	31.09	817.52	905.38	393.62	340.01	91.98	46.87	96.12	45.75
	**sd**	7.76	7.75	7.10	7.19	0.056	0.056	0.044	0.048	8.52	7.63	6.33	7.17	0.038	0.101	0.037	0.070
**Combine DC + ED + LC**	**mean**	19.56	16.22	18.51	13.90	5.77	2.12	5.72	2.14	20.21	16.48	18.73	13.53	5.69	2.12	5.76	2.14
	**sd**	6.80	3.04	6.09	4.91	0.036	0.021	0.027	0.014	7.06	3.51	6.27	5.35	0.033	0.020	0.025	0.017
**Total**		**59.69**	**56.41**	**60.88**	**56.06**	**36.89**	**33.25**	**36.80**	**33.23**	**837.73**	**921.85**	**412.35**	**353.54**	**97.67**	**48.98**	**101.88**	**47.89**
